# Clinical Recollections

**Published:** 1896-02-01

**Authors:** Benjamin Ward Richardson


### Clinical Recollections.

By Sir Benjamin Ward Richardson, M.D., F.R.S.

In my previous papers in The Hospital I have published articles of various kinds, but particularly some on anæsthesia and anæsthetics. I propose now, under the present head, to bring together various recollections of the past in their bearings on the practice of medicine. I have been singularly well stored in this direction of thought for a long series of years, and have kept, throughout, notes of all cases that were of particular interest and on which I have expended special attention. It may, therefore, be of interest to members of the profession who are studying the same class of cases to read what has occurred to me, and sometimes to be guided, perhaps, by the lessons I have learned, or the inferences I have drawn. Not infrequently the observations I shall make have been derived from the study of hospital cases, for I have been three times in my life connected with hospital practice. At other times the study will be from cases of a private kind derived from the clinique that has been before me in consultation, or from the patients who have come to consult me in morning home work—always a class to be carefully noted—in whom diagnosis is the leading feature, but for whom treatment and mode of life call for the most serious consideration.

### Alcohol in its Character as an Aid in Practice.

It occurs to me at this moment to commence to deal with a clinical study by taking up alcohol as the greatest subject that now lies before the profession for its judgment. Alcohol has at all times had two aspects in so far as we medical men are concerned. We have thought of it as a food, and have wondered whether we are or are not wise in administering it in that sense. Some have conceived that, as a food, it performs certain definite functions. Many think that it helps to build up parts of the body, and is, in the strictest sense, a nutritive food; it may, or may not, have those qualities, but that it supplies, in some as yet unexplained manner to their minds, warmth to the body, and in that sense gives what is really necessary for the maintenance of life they do not doubt. A third set think that alcohol acts after the manner of an anæsthetic, that it obliterates pain; and the first claimant, I believe, of the discovery of general anæthesia affirmed that he originally noticed how alcohol narcotised—to such an extent that an operation could be performed under it painlessly—simply by his having seen the fumes of alcohol inhaled during an accident. To such an argument may also be added a statement, which I recollect from the earliest days, that alcohol is a direct anæsthetic or soother of particular nervous pains, such as neuralgia pains—brow ague and toothache—the action under these circumstances being on, and through, the nervous system.

There has also been a very large school which has looked upon alcohol as an agent having the power of stopping waste of tissue, and which has administered it for the reason that it suspended the influence of causes which were leading to direct destruction of the body by what may be called elimination through the skin, through the lungs, through the kidneys, through the intestines, and through the glandular system generally. Lastly, there has been a school which may be really considered universal in its character, which has held up alcohol as a stimulant, and has assumed that whenever the vital powers were failing there was always actual need for alcoholic administration. The members of this school live yet in large numbers both within and without the profession, and testify to their belief by that which they undertake in their acts. They see a man or a woman become faint from some cause or other, and they fly to the nearest form of alcohol as the stimulant, pour it in copiously sometimes, and consider that the effect is invaluable; or they see a man or a woman collapsed and wearing out from disease, and again they fly to alcohol as the stimulant, and give it for the purpose, as they believe, of retaining life, ignoring altogether the circumstances under which life is decaying, as well as all the results which may spring from the alcohol itself, and which are exceedingly serious in a vast number of instances.

In casting a survey over these schools, some of which are of modern growth, I fail to see anything like unanimity in regard to the form in which the alcohol is employed. Anciently, the particular word "alcohol" could not have been used, but what was commonly called "wine" was the thing given. "Give wine unto him that is about to perish"—although the saying merely referred originally to those who were going to pass through a painful death, and to whom wine would rather be a stupefier than anything else—did undoubtedly convey a great deal, and, as if in a sentence, suggested the general principle that wine was necessary for the maintenance of life. The fathers of physic used the word always in this way, for when we turn to them and gather their exact phraseology, "wine," it seems, was the word always employed, as if they knew—in the far-off days—that no other word could properly express their meaning, and as if they thought that wine itself was a distinct thing, having a definite name as well as a definite purpose. I believe that to the present hour the term "wine" has, by the majority of people the same meaning; for, we are often asked, "Would not a little wine give strength or relief?" and when we explain that wine is nothing more than alcohol mixed with a certain measure of water, we are not understood in the right sense of the word.

By these teachings we have seemed to speak of wine as if it were a distinct thing, as the older men did; but in the present day we have to study, not wine alone, but all fluids containing alcohol—wine, brandy, gin, rum, beer, or any alcoholic fluid. We have swerved from the old starting-point, whether we consider the question from the feeding or the medicinal side, and, curiously enough, we often swing into contradictions on the same article merely by a verbal distinction. One man would give beer or stout that the body taking it may be kept up; but the same man, in precisely the same case, would not give brandy or gin, because neither would keep up; yet all the time through it was one thing that was given, or supposed to be given, for a specific purpose, and that was the alcohol.

